# *In vivo* monitoring of hair cycle stages via bioluminescence imaging of hair follicle NG2 cells

**DOI:** 10.1038/s41598-017-18763-3

**Published:** 2018-01-10

**Authors:** Yasuhisa Tamura, Kumi Takata, Asami Eguchi, Yosky Kataoka

**Affiliations:** 1 Cellular Function Imaging Team, RIKEN Center for Life Science Technologies, 6-7-3 Minatojima-minamimachi, Chuo-ku, Kobe, 650-0047 Japan; 2 Multi-Modal Microstructure Analysis Unit, RIKEN CLST-JEOL Collaboration Center, 6-7-3 Minatojima-minamimachi, Chuo-ku, Kobe, 650-0047 Japan

## Abstract

Hair growth occurs periodically in a cycle that consists of three different phases: growth, regression, and resting. The length of each phase is regulated by both intrinsic and extrinsic factors throughout life, and influenced by physiological and pathological conditions. Elongation of the resting phase and shortening of the growth phase occur during physiological ageing and in baldness, respectively. *In vivo* discrimination of each phase of the hair cycle can be used to research for regeneration of hair follicles as well as to evaluate the efficacy of hair regrowth treatments in the same individual. Here we show that NG2+ epithelial cells in the hair follicles encompass bulge stem cells, and that the number of hair follicle NG2 cells underwent dramatic changes during the hair cycle. Transgenic rats with expression of firefly luciferase gene in NG2 cells were generated to monitor the hair cycle *in vivo*. Hair follicle NG2 cells were clearly visualized via bioluminescence imaging to study each phase of the hair cycle in the rats, from infancy to old age.

## Introduction

During postnatal life, hair follicles are periodically regenerated through a continuous cycle that comprises three distinct phases: anagen (growth phase), catagen (regression phase), and telogen (resting phase)^1,2^. At the beginning of the anagen phase, stem cells present in the bulge region of the hair follicle proliferate and generate outer root sheath (ORS) cells. Prior to activation of hair follicle stem cells, secondary hair germ (sHG) cells rapidly proliferate and produce the transit amplifying cells (TACs) in the germinal matrix. Subsequently, the TACs differentiate into inner root sheath (IRS) cells and hair shafts^3,4^. During catagen, cells in the ORS and the germinal matrix undergo apoptosis, and the lower two-thirds of hair follicles regress. In telogen, hair follicle stem cells are relatively quiescent and the bulge of the hair follicles is in contact with the dermal papilla. Therefore, these stages may be distinguished according to hair follicle morphology and cellular behaviour via histological studies; however, this requires the preparation of skin sections from biopsy samples. In rodents, skin pigmentation is used as classical methods for studying hair cycle stages based on the monitoring of melanogenesis, which starts at early-to-mid anagen^5^. It is difficult to accurately evaluate hair follicle growth stages and to quantitatively monitor cellular dynamics of hair follicles using this method. Recently, the development of imaging methods for monitoring the hair cycle, using *in vivo* bioluminescence imaging, has been reported^6^. These imaging techniques enable clear discrimination of each hair cycle stage on the basis of wingless-type mouse mammary tumour virus integration site (Wnt)/β-catenin signalling activity during hair regeneration in living mice. Wnt signalling plays a crucial role in hair follicle morphogenesis and regeneration^7,8^. Therefore, this signalling pathway was considered a suitable target for monitoring hair growth phases. However, the timing of entry into the anagen phase is also regulated by other signalling molecules than Wnt, including bone morphogenetic protein (BMP), Sonic hedgehog (Shh), fibroblast growth factor (FGF), and transforming growth factor (TGF)-β. These molecules work under different conditions; Shh promotes entry into anagen^9–11^ while TGF-β facilitates the initiation of anagen in the hair follicle cycle^12^. That brings about difficulty to monitor the hair cycle states only via the expression dynamics of single molecule. In addition, activation of the Wnt/β-catenin pathway failed to report the stimulation of hair growth with minoxidil, a drug utilised for treatment of hair loss and baldness by promoting telogen-anagen transition^13^. Thus, we established an *in vivo* imaging method for monitoring hair cycle stages based on the morphological change of the hair follicles. During the progression of the hair cycle, the upper third of the hair follicle remains almost unchanged; however, the lower two-thirds of the hair follicle undergoes drastic morphological changes resulting from cell proliferation and the apoptosis of stem cells and their progeny in the hair follicles. In rodents, hair follicle stem cells and their progeny are mainly found in the bulge and ORS. In addition, sHG cells and their progeny are present in the germinal matrix. It has been reported that many cells in the bulge region, the ORS, and the sHG of hair follicles express NG2 (chondroitin sulphate proteoglycan 4; CSPG4) in postnatal mice^14^ and humans^15^. Therefore, *in vivo* imaging of hair follicle NG2-expressing cells (NG2 cells) should enable monitoring of the hair cycle based on the morphological dynamics.

In this study, we demonstrated that NG2 cells in the hair follicles represent hair follicle epithelial cells and dermal papilla cells in adult skin, and that only NG2-expressing hair follicle epithelial cells exhibited proliferative activity. Moreover, we generated transgenic (Tg) rats expressing firefly luciferase (fLuc) gene under the control of the rat NG2 promoter (NG2-fLuc), in order to visualize NG2 cells in the hair follicles *in vivo*. Using the NG2-fLuc Tg rats, we succeeded in monitoring hair cycle from infancy to aging.

## Results

### Characterization of NG2 cells in the hair follicles of adult rats

**Figure 1:**
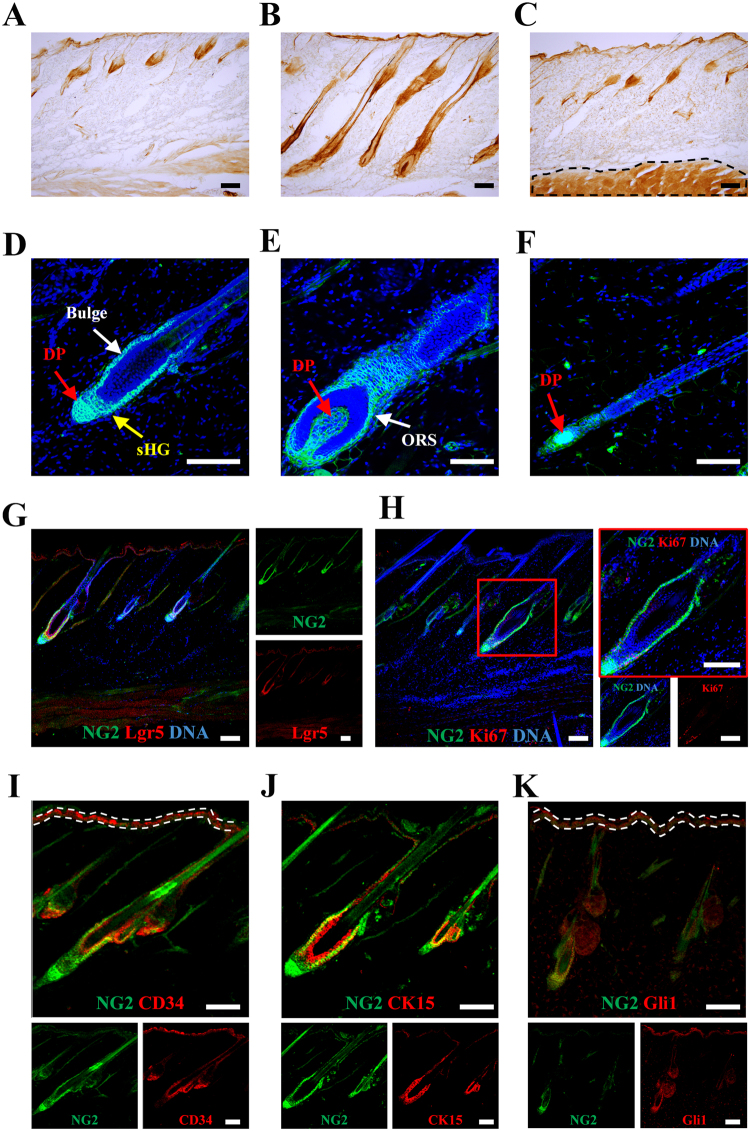
Localization and characterization of NG2 cells in the hair follicles of adult rats during each phase of the hair cycle. (**A**–**C**) Localization of NG2 cells in the hair follicles during telogen (**A**), anagen (**B**), and catagen phase (**C**). (**C**) The immunoreactivity in the muscle (shown by dashed line) was non-specific binding of the secondary antibody. (**D**–**F**) Immunofluorescence images showing staining for NG2 (green) and DNA (blue) during telogen (**D**), anagen (**E**), and catagen phases (**F**); sHG, secondary hair germ; DP, dermal papilla; ORS: outer root sheath. (**G**) Expression of Lgr5 (red) in NG2 hair follicle epithelial cells (green) of adult rats. (**H**) A proportion of the hair follicle NG2 cells enter the cell cycle during anagen phase; double immunofluorescence staining for NG2 (green) and Ki67 (red), a marker for cells entering the cell cycle, in the hair follicles of adult skin: right panels show magnified views of the red square in the left panel. (I–K) A proportion of NG2 cells represent CD34-, cytokeratin 15 (CK15)-, and/or Gli1-expressing hair follicle stem cells in the bulge of the hair follicle. Skin sections stained with NG2 (green) and CD34 (red in I), CK15 (red in J) or Gli1 (red in K) antibodies, which are markers for hair follicle stem cells. (**I** and **K**) The immunoreactivity in the interfollicular epidermis (shown by dashed white line) was non-specific binding; scale bars, 10 μm (**D**–**F**), 100 μm (**G**–**K**), and 200 μm (**A**–**C**).

NG2 cells are present in the bulge and dermal papilla of early postnatal mice^14^. In the adult skin, NG2-immunopositive (NG2) cells were mainly observed in hair follicles, and the number of NG2 cells varied at each phase of the hair cycle (Fig. 1A–C). At the telogen phase, NG2 cells were found in the bulge, sHG, and dermal papilla in normal Wistar rats (Fig. 1D). Further, NG2 cells were observed in the outer root sheath (ORS) during the anagen (Fig. 1E) and catagen phases (Fig. 1F). Lgr5-expressing cells are found in the bulge, sHG, and ORS of hair follicles, and represent hair follicle stem cells and their progeny^16^. Double-immunofluorescence staining showed that almost all NG2 cells were immunopositive for Lgr5 in the bulge, sHG, and ORS during the hair cycle; however, NG2 cells in the dermal papilla did not exhibit Lgr5 immunoreactivity (Fig. 1G). Based on the localization and antigenicity of NG2 cells, the Ki67 antibody, a marker for proliferating cells, was used to investigate whether NG2 cells exhibit proliferative ability. Some NG2 cells in the bulge, sHG, and ORS were also immunoreactive for Ki67 (Fig. 1H). A proportion of NG2 cells in the bulge were also immunopositive for CD34^17^, CK15^18^ or Gli1^19,20^, which are markers for hair follicle stem cells (Fig. 1I–K). These data suggest that NG2 cells in the hair follicles are composed of two kinds of cell populations: an epithelial cell compartment (including hair follicle stem cells in the upper, middle, and lower bulge), and a stromal compartment (i.e. dermal papilla cells) in the adult rats (Fig. 1G–K), and that the number of hair follicle NG2 cells dramatically changes during the hair cycle (Fig. 1A–C). In this study, we demonstrated *in vivo* monitoring of the hair cycle by NG2 expression.

### Generation of transgenic rats

In order to generate rats expressing firefly luciferase in the NG2 cells, we prepared bacterial artificial chromosome (BAC) transgenic (Tg) rats expressing firefly luciferase (fLuc) in NG2 cells. The BAC constructs were microinjected into fertilized eggs of Wistar rats, and the fertilized eggs were implanted into pseudopregnant Wistar rat females for producing the NG2-fLuc Tg rats^21^. In the obtained NG2-fLuc Tg rats, one out of three founders showed a slightly weaker bioluminescence signal by application of luciferin, and one of the remaining two founders did not inherit the BAC transgene. The line derived from the third founder was used for further studies. The offspring of the Tg rats were identified via polymerase chain reaction (PCR) analysis of genomic DNA obtained from ear skin (Supplemental Fig. S1A).

### Expression of the fLuc gene in hair follicle NG2 cells of the transgenic rats

**Figure 2:**
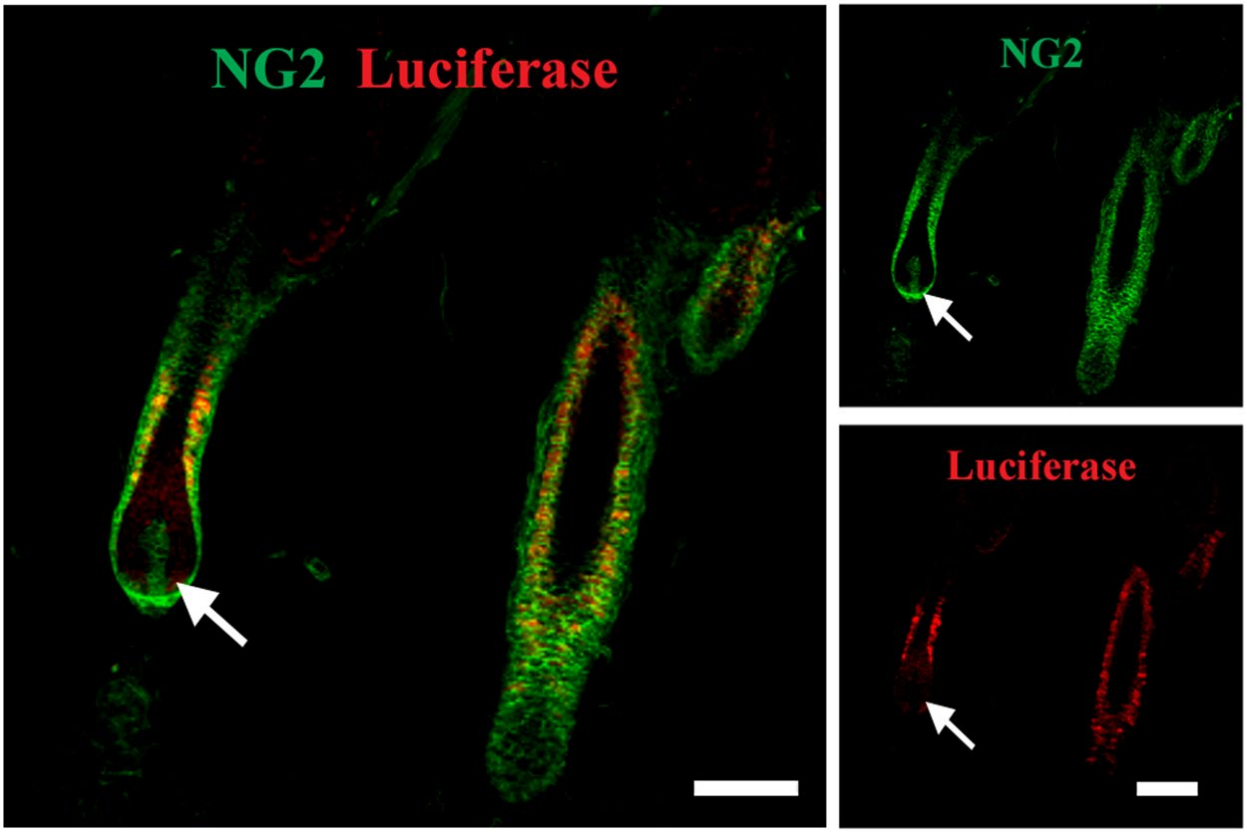
Expression of fLuc protein in the hair follicle NG2 cells of Tg rats; skin sections from Tg rats stained with NG2 (green) and luciferase (red) antibodies. Arrows indicate dermal papilla cells that are immunopositive for NG2, but not for luciferase; scale bars; 100 μm.

In order to examine whether the fLuc gene is expressed in the skin of the NG2-fLuc Tg rats, we isolated mRNA from dorsal skin as well as visceral tissues in the abdomen and performed real-time PCR. fLuc gene expression was observed in the skin of the adult Tg rats, but not that of the WT rats; the expression level in the skin was similar to that in the stomach and bladder in both male and female Tg rats (Supplemental Fig. S1B). The fLuc gene appeared to be expressed in the other tissues, including the kidney and intestinal tract (Supplemental Fig. S1B). These data were confirmed by analysis of the gene expression of NG2 in each tissue of Tg rats (Supplemental Fig. S1C). Furthermore, immunohistochemical staining using anti-luciferase and anti-NG2 antibodies showed that fLuc expression was observed in almost all NG2-expressing epithelial cells in hair follicles, but not in the NG2-expressing dermal papilla cells (Fig. 2).

### Bioluminescence imaging of hair follicle NG2 cells during the first postnatal hair cycle

**Figure 3:**
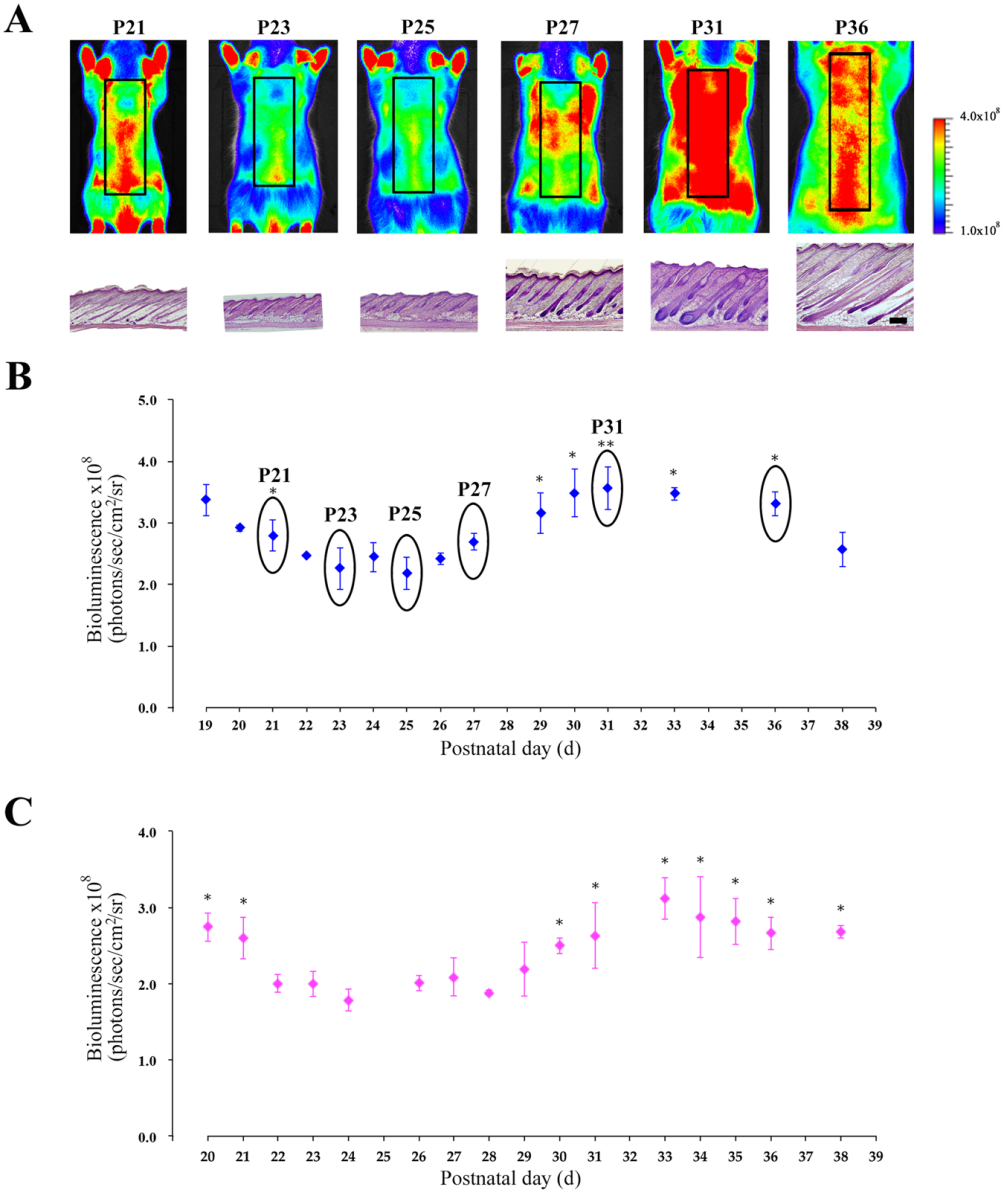
*In vivo* bioluminescence imaging of NG2-fLuc Tg rats during the first postnatal hair cycle. (**A**) Bioluminescence images of male Tg rats from P19 to P38 during the first postnatal hair cycle; black squares in each image show regions of interest (ROI). Haematoxylin-stained images of skin from Tg rats visualized by bioluminescence imaging at each time point; scale bars; 500 μm. (**B** and **C**) Quantification of bioluminescence signals from ROI in the dorsal surface skin of male (N = 14, blue diamonds in **B**) and female (N = 8, pink diamonds in **C**) Tg rats during the first postnatal hair cycle. Data from each animal were presented as means ± SD. *P < 0.05 and **P < 0.01; compared with telogen phase (P23 in B or P24 in **C**).

Morphogenesis of hair follicles is complete within 2 weeks after birth in mice^5^. Initially, we performed bioluminescence imaging using the Tg rats from postnatal days 12 (P12) to P18 during hair follicle morphogenesis, as the intravenous injection of luciferin in infant rats (before P12) was challenging. At P16, bioluminescence signals from the dorsal skin were clearly visualized in the Tg rats, but not in the WT rats (Supplemental Fig. S2A). *In vivo* imaging revealed bioluminescence signals in the skin as well as in the internal organs, including the stomach, intestinal tract, and bladder, of Tg rats (Supplemental Fig. S2B). Furthermore, *ex vivo* imaging obviously showed that signals were detected in the stomach, intestinal tract, and bladder, as well as in the kidney, but not in the liver and spleen, (Supplemental Fig. S2C). Bioluminescence signals from the skin were possibly contaminated with the signals from the internal organs, as the skin of rats was very thin during hair follicle morphogenesis. In order to clarify signal contamination, we performed bioluminescence imaging of skin NG2 cells with or without masking tapes, which were used to block bioluminescence signals emitted from the other organs (Supplemental Fig. S2D). When all internal tissues in the abdomen were wrapped with masking tape, bioluminescence signals from the skin were about 10% lower than those from the skin of rats without masking tape at P16-P17 (Supplemental Fig. S2D and E). At P19, both groups showed similar levels of the bioluminescence signals from the skin in male Tg rats (Supplemental Fig. S2E). Moreover, in female Tg rats, the bioluminescence signals from the skin could be detected without contamination of signals from the internal organs at P20 (Supplemental Fig. S2E). Following morphogenesis, hair follicles enter the first postnatal hair cycle: catagen at P16, telogen at P22, and anagen at P28. During this period, hair follicles are synchronized^5^. Therefore, we performed bioluminescence imaging studies using Tg rats from first catagen (P19: male or P20: female) to second catagen (P38). In male Tg rats, bioluminescence signals from NG2 cells decreased from P19 to P23, and were maintained at a similar level from P23 to P25, corresponding to telogen to early anagen phases (Fig. 3A,B). The signals began to increase at P26, and gradually increased until P31; after this stage, the bioluminescence signals decreased (Fig. 3B). In female Tg rats, bioluminescence signals were observed at basal levels for 5 days, from P24 to P29; the signal intensity began to rise at P30, and then gradually increased until P33 (Fig. 3C). In order to confirm the bioluminescence imaging data, we performed haematoxylin staining of skin sections from each animal. Staining studies showed that almost all hair follicles in the skin of Tg rats at P23, P25, and P31 were in the telogen, early-anagen, and mid-anagen phase, respectively^5^. Therefore, the imaging data was supported by the results of section staining experiments at each time point (Fig. 3A). Moreover, in female Tg rats, the onset of anagen was delayed for a few days relative to male Tg rats (Fig. 3B and C). This finding has been reported by previous studies in mice^6^.

### Bioluminescence imaging of hair follicle NG2 cells during depilation-induced hair cycle

**Figure 4:**
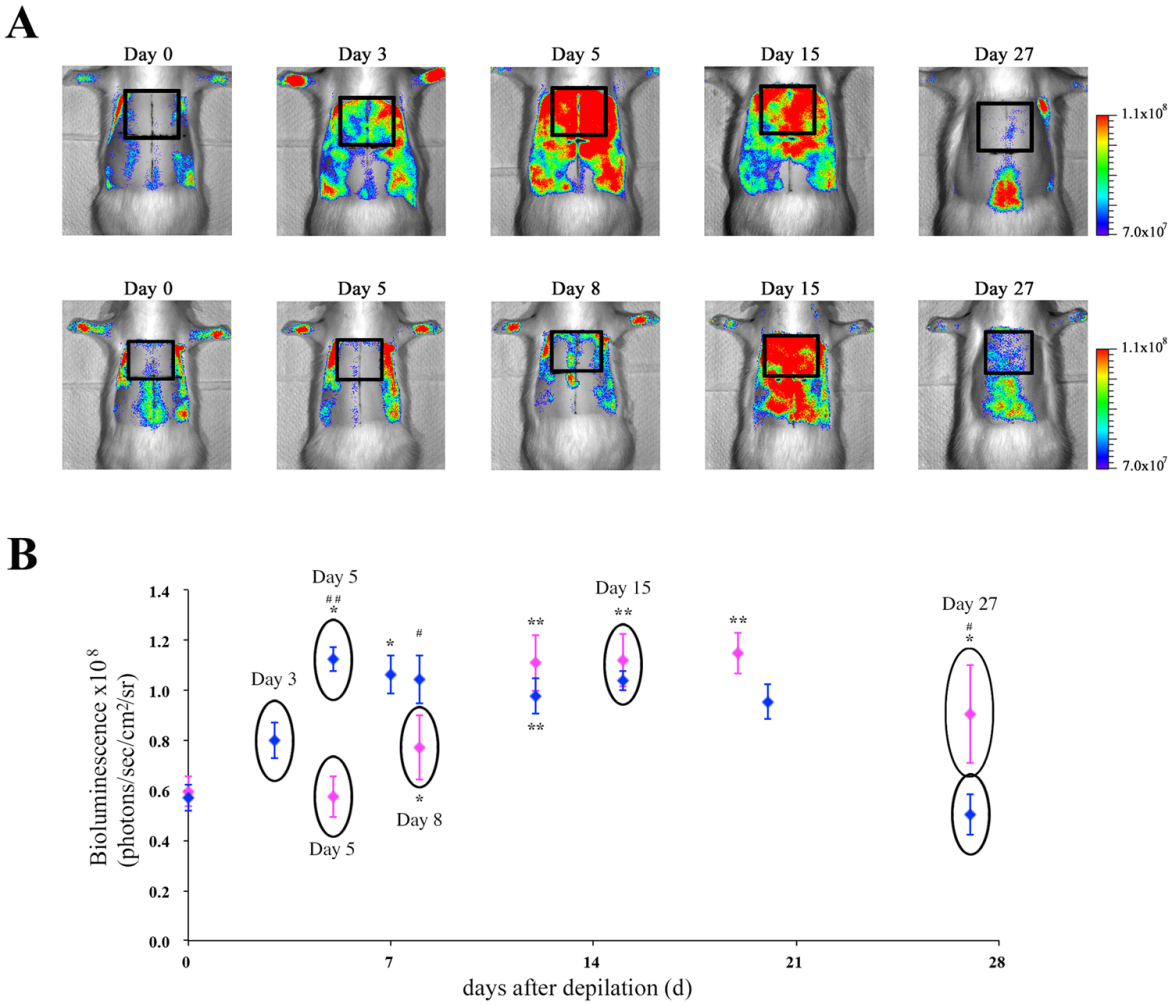
*In vivo* bioluminescence imaging of hair follicle NG2 cells during depilation-induced hair cycle in NG2-fLuc Tg rats. (**A**) Bioluminescence images of male (upper panels) and female (lower panels) Tg rats during depilation-induced hair cycle; black squares in the bioluminescence images show ROIs. (**B**) Quantification of bioluminescence signals from ROIs in the dorsal surface skin of both male (N = 9, blue diamonds) and female (N = 7, pink diamonds) Tg rats at each time point after depilation. Data from each animal were presented as means ± SD. *P < 0.05 and **P < 0.01; compared with pre value (Day 0). ^#^P < 0.05 and ^##^P < 0.01; compared between male and female Tg rats at each time point.

**Figure 5:**
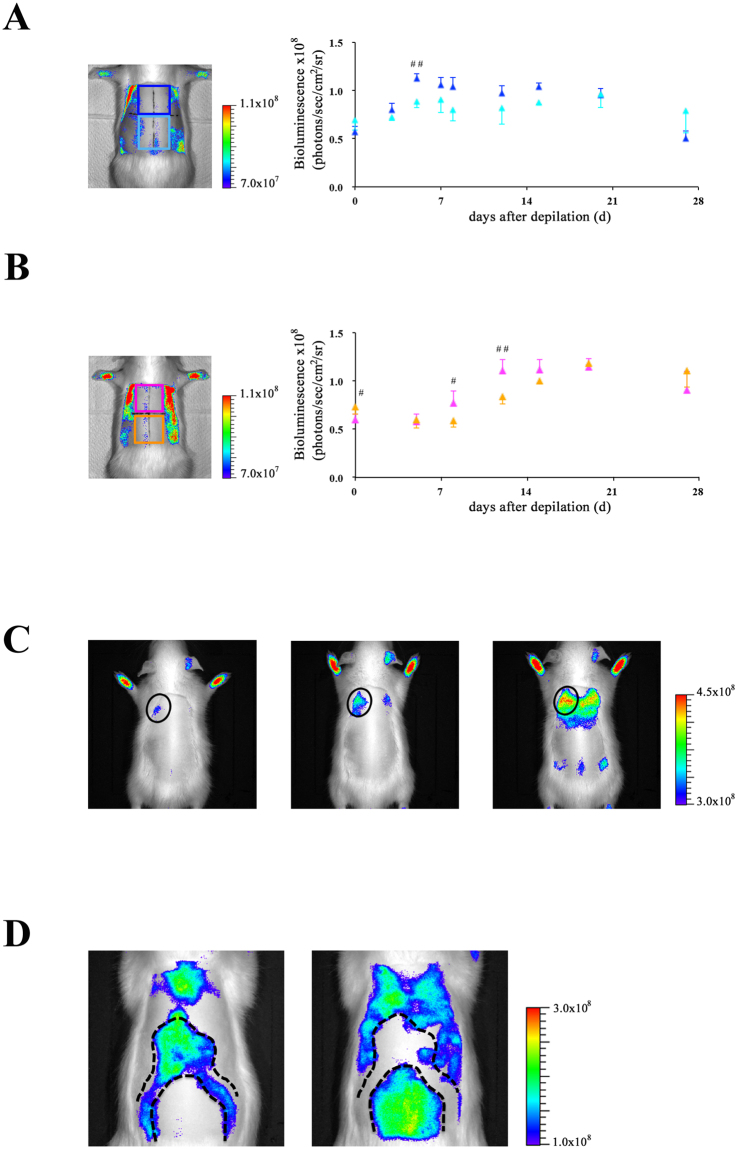
*In vivo* detection of regenerative domain patterns of hair cycling via bioluminescence imaging of hair follicle NG2 cells. (**A** and **B**) *In vivo* detection of propagating anagen waves via bioluminescence imaging of hair follicle NG2 cells during depilation-induced hair cycle. *In vivo* bioluminescence imaging of dorsal skin in male (**A**) and female (**B**) Tg rats at each time point after depilation; each ROI (upper region and lower region along the rostrocaudal direction on the dorsal skin) is shown as blue and light blue squares (**A**) or pink and orange squares (**B**), respectively; quantification of bioluminescence signals from upper regions (blue in A or pink triangles in **B**) and lower regions (light blue in A or orange triangles in **B**) in the dorsal skin of male (N = 9) and female (N = 7) Tg rats. Data from each animal were presented as means ± SD. ^#^P < 0.05 and ^##^P < 0.01; compared between upper and lower regions in the skin at each time point. (**C** and **D**) Bioluminescence imaging of hair follicle NG2 cells enables detection of hair cycle domain patterns, including initiation events (black circles in **C**), and border stability (back dashed line in **D**). (**D**) Subpopulations of hair follicles in separated regions by dashed lines were in the different stages of hair cycle. These images were obtained from Tg rat skin at postnatal days 72 (left panel) and 91 (right panel).

After 6 weeks, hair cycling becomes gradually less synchronized as aging occurs^22^. However, synchronicity of hair cycling may be revived by depilation of hair shafts in the telogen phase^5^. We investigated whether the depilation-induced hair cycle may be visualized by *in vivo* imaging of the Tg rats. In male Tg rats, the bioluminescence signals from the dorsal skin were observed at maximal levels at 5 days after depilation. These signals were maintained at the same level over the next 2 weeks, following which the signals declined (Fig. 4A and B). In contrast, in female Tg rats, the bioluminescence signals were detected at basal levels within 5 days; these then gradually increased until 12 days. Furthermore, the signals were detected at maximal levels for 1 week; these then began to decrease within a few days (Fig. 4A and B). Similar to that in the first postnatal hair cycle, in the depilation-induced hair cycle, the onset of entry into anagen in female rats was later than in male rats. Moreover, bioluminescence imaging studies showed that the onset of entry into anagen in the rostral regions preceded that in the caudal regions in dorsal skin of both male and female rats (Fig. 5A and B). This phenomenon is known as the propagation of the anagen wave^23^. In addition, we investigated whether this imaging technique enables detection of regenerative domain patterns of hair cycling, including initiation events and border stability^23,24^. The initiation event is the process that a small population of hair follicles starts to spontaneously enter the anagen phase from the telogen phase, and then, the anagen phase spreads to the surrounding areas. Border stability is the phenomena that the hair cycle domains in between neighboring areas are entering in different phases of hair cycle. As shown in Fig. 5C and D, these phenomena could be visualized in the hair follicle NG2 cells *in vivo* using the present imaging method. These findings indicate that bioluminescence imaging of hair follicle NG2 cells will be used to monitor the hair cycle in infant and adult rats.

### *In vivo* monitoring of the spontaneous hair cycle in aged rats via bioluminescence imaging of hair follicle NG2 cells

**Figure 6:**
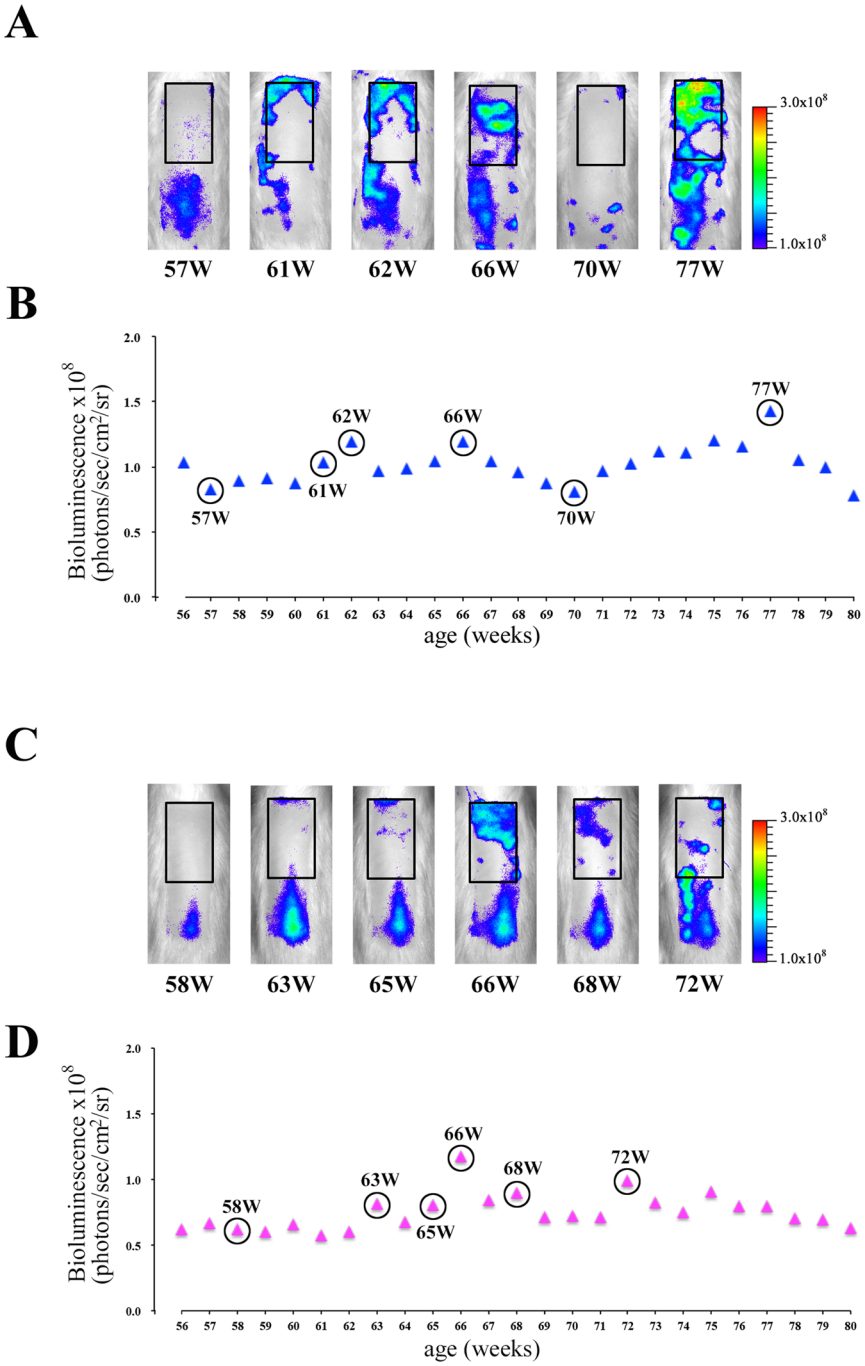
*In vivo* monitoring of spontaneous hair cycle in aged rats via bioluminescence imaging of hair follicle NG2 cells. Typical bioluminescence imaging data of male (**A** and **B**) and female (**C** and **D**) aged Tg rats (56- to 80-week-old). (**A** and **C**) Bioluminescence images of each Tg rat; black squares show regions of interest (ROI). ROIs were defined on the upper region of the dorsal surface skin in each aged rat as that in young rats in Fig. 4A. (**B** and **D**) Bioluminescence signals from ROIs in the dorsal surface skin of both male (blue triangles in **B**) and female (pink triangles in **D**) aged rats.

**Figure 7:**
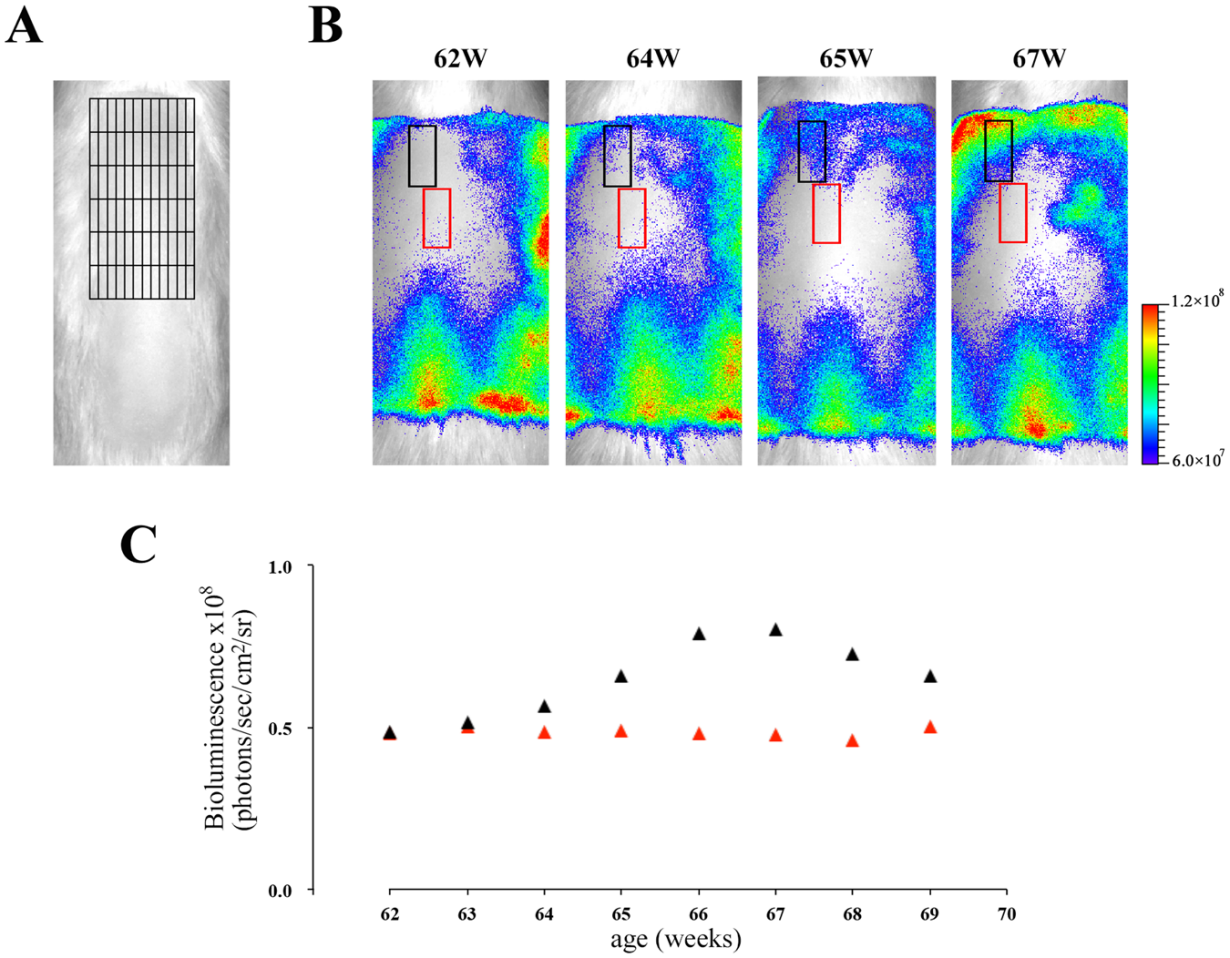
*In vivo* detection of the dynamics of fragmented hair cycle domains in aged Tg rats. (**A**) Representative image of a 12 × 6 grid in defined rectangular regions (in Fig. 6A and C) of aged Tg rat skin. The defined region was divided into 72 areas with the grid (black lines). Each area: 1.5 mm × 5.5 mm (8.25 mm^2^). (**B**) Bioluminescence images of aged Tg rat; each square with black or red lines shows regions of interest (ROI). Each ROI was composed of 6 areas (6 × 8.25 mm^2^). (**C**) Bioluminescence signals from each ROI with black or red lines in B are shown with black triangles or red triangles during 62–69 weeks old, respectively.

*In vivo* bioluminescence imaging was performed in male (N = 4) and female (N = 7) aged Tg rats during 56–80 weeks old. Typical imaging data in male and female aged Tg rats were shown in Fig. 6A,B and C,D, respectively. The alterations of bioluminescence signals in aged Tg rats (Fig. 6) were different from those in infant and young Tg rats (Figs 3 and 4). These data suggested that the hair follicles in aged skin entered in the anagen phase individually and asynchronously in contrast to young rats. Indeed, these phenomena have been known as the fragmentation of hair cycle domains in aged mice skin^25^. Moreover, we performed a more detailed analysis of the imaging data with a 12 × 6 grid in order to detect the dynamics of fragmented hair cycle domains (Fig. 7A). The detailed analysis showed that both re-entry into the anagen phase and telogen retention of each small hair cycle domain can be clearly detected in aged Tg rats (Fig. 7B and C). In aged mice skin, the elongation of the telogen phase was also observed^25^. We investigated the duration period of telogen phase in both male (N = 4) and female (N = 7) aged Tg rats by using this imaging data. The retention period of the telogen phase was 34–115 days in aged Tg rats. Moreover, the duration of telogen phase in male and female aged rats was 62 ± 27 (34–99 days) and 93 ± 23 (47–115 days) days, respectively. These data suggested that telogen retention in female aged rats tended to be longer than that in male Tg rats. These imaging data were confirmed by the histological staining study of skin sections obtained from aged Tg rats (Supplemental Fig. S5). These data indicated that the present imaging technique can be used to detect the dynamics of hair cycle during all the life span of animals.

## Discussion

The hair cycle consists of three different phases: the growth (anagen) phase, the transitional (catagen) phase, and the resting (telogen) phase. *In vivo* imaging of each stage of the hair cycle is useful for basic research into hair cycle, as well as for screening hair growth-promoting agents. To date, there have been few reports of *in vivo* monitoring techniques for the hair cycle based on skin pigmentation studies^5^ and bioluminescence imaging of the Wnt signalling pathway^6^. Quantitative analysis of the cellular dynamics of hair follicles, during the hair cycle, is challenging to achieve *in vivo* via skin pigmentation studies. Recently, *in vivo* methods for monitoring the hair cycle based on bioluminescence imaging of the Wnt/β-catenin signalling pathway have been established^6^. However, the Wnt/β-catenin signalling pathway is involved in the proliferation of hair follicle stem cells and their progeny, as well as of dermal fibroblasts and adipocyte stem/progenitor cells in the skin^26,27^. It has been reported that minoxidil stimulates hair regrowth without activation of the Wnt/β-catenin signalling pathway^13^. Therefore, the development of *in vivo* imaging techniques for monitoring hair cycle stages, based on hair follicle morphology, are necessary. In this study, we succeeded in establishing a method for *in vivo* monitoring of the hair cycle via bioluminescence imaging of hair follicle NG2 cells. Indeed, this imaging technique can monitor the spontaneous hair cycle in infant rats (Fig. 3) as well as the depilation-induced hair cycle in young Tg rats (Fig. 4). In aged Tg rats, elongation of telogen phase and fragmentation of hair cycle domains can be also clearly detected (Fig. 5). These phenomena in aged rodents were supported by previous findings from skin pigmentation studies^25^.

NG2 cells are found in the bulge, sHG, and ORS during the hair cycle^14,15^. In addition, Lgr5-expressing cells are found in the bulge and sHG at the telogen phase and in the ORS during the anagen phase^16^. However, the relationship between NG2 cells and Lgr5-immunopositive (+) cells in the hair follicles is poorly understood. The present double-immunostaining data indicated that NG2 cells in the hair follicles are composed of Lgr5(+) hair follicle epithelial cells in the sHG and ORS, and Lgr5(−) cells in the dermal papilla (Fig. 1). In the present NG2-fLuc Tg rats, expression of fLuc protein was observed in the NG2(+)/Lgr5(+) sHG and ORS cells, but not in the NG2(+)/Lgr5(−) dermal papilla cells (Fig. 2). Recently, NG2 expression was observed in the dermal adipocytes of mice^28^. In the present Tg rats, NG2(+) adipocytes of the dorsal skin did not exhibit fLuc expression (data not shown). These findings suggest that the bioluminescence signals originating from the skin were emitted only from NG2(+)/Lgr5(+) epithelial cells in the hair follicles. Therefore, the present NG2-fLuc Tg rats may be used to monitor the cellular dynamics of hair follicle NG2 cells.

In the mammalian brain, NG2 cells proliferate and produce mature oligodendrocytes during development^29^ and adulthood^30^. In the adult brain, NG2 cells additionally produce astrocytes or neurons under physiological and pathological conditions^31–34^. These findings indicate that NG2 cells represent progenitor cells in the brain. Furthermore, NG2(+) cells may additionally function as progenitor cells in the hair follicles. Accordingly, in the present work, NG2(+) cells showed immunoreactivity for Ki67 in the hair follicles (Fig. 1G). Moreover, almost all NG2 cells were immunoreactive for Lgr5 in the sHG and the ORS of the hair follicles (Fig. 1H). Hair follicle Lgr5-expressing cells proliferate during the anagen phase and maintain all cell types of the hair follicles, as shown via cell lineage-tracing experiments^16^. These findings indicate that hair follicle NG2 cells are multipotent, with the ability to differentiate into a variety of cell types in hair follicles.

In the present imaging studies, bioluminescence signals from NG2 cells were detected in the intestinal tract, stomach, and bladder, as well as in the skin (Supplemental Fig. S3B). Bioluminescence signals from each internal organ appeared to correspond to the expression of the fLuc gene in Tg rats (Supplemental Fig. S2B). Thus, bioluminescence signals from these tissues seem likely to spill over into those from skin in *in vivo* bioluminescene imaging. However, we showed that bioluminescence signals from skin were not affected by those from these internal tissues after P19 in male and P20 female Tg rats (Supplemental Fig. S3D and E). Among these tissues, the presence of NG2 cells in the intestinal tract and kidney has been supported by previous reports^35,36^. *Ex vivo* studies indicated that the bioluminescence signals from NG2 cells in the kidney were weaker than those from the other internal organs, including the stomach, intestinal tract, and bladder (Supplemental Fig. S3C). Indeed, expression of fLuc and NG2 mRNA was detected in the kidney of Tg rats (Supplemental Figs S2B and S3C). However, it is not clear why bioluminescence signals from NG2 cells could be only faintly detected in the kidney relative to the other internal organs. Notably, to our knowledge, the existence of NG2 cells in the stomach has not been reported to date. Therefore, the present imaging method has revealed, for the first time, the possible presence of NG2 cells in the stomach of adult rats.

Tg rats used in this study were generated for *in vivo* dual-modality imaging of NG2 cells. These rats expressed fLuc and the human oestrogen receptor ligand binding domain (hERL) in NG2 cells, for bioluminescence and positron emission tomography (PET) imaging, respectively^37^. The hERL, which is a fragment of the oestrogen receptor, possesses a binding site for oestrogen or its analogues, including 16α-[^18^F]-fluoro-17β-estradiol (^18^F-FES)^38^. In the Tg rats, endogenous oestrogens bind oestrogen receptors as well as hERL; this may have affected the levels of endogenous oestrogens. This has led to concerns that Tg rats cannot be used for monitoring spontaneous and induced hair cycles, as the hair cycle is regulated by oestrogens^39–41^. However, when hair follicle morphology in each animal was compared, no difference in hair cycling was observed between Tg and normal Wistar rats until at least 8 weeks of age. Therefore, the expression of the hERL gene is considered to have little effect on hair cycling in the Tg rats.

Here, we showed that NG2 cells may represent a population of hair follicle progenitor cells. In addition, we demonstrated that the present method, which is based on bioluminescence imaging of hair follicle NG2 cells, may be utilized to monitor hair cycling from infancy to adulthood *in vivo*. However, this imaging technique has some concerns. In this study, it cannot absolutely be excluded the possibility that bioluminescence signals from hair follicle NG2 cells are contaminated with the signals from the NG2-expressing cells in the internal organs under several conditions. In addition, the roles of NG2 cells in hair follicle biology and pathology remain to be fully elucidated. Therefore, it needs attention when this imaging method is used for monitoring of hair cycle dynamics under physiological and pathological conditions. Further studies are necessary in order to solve the problems.

## Methods

### Generation of transgenic rats

Transgenic (Tg) rats were obtained from PhoenixBio (Utsunomiya, Japan). The rat NG2 (chondroitin sulphate proteoglycan 4; CSPG4) bacterial artificial chromosome (BAC) clone, CH230-428C7, was selected from the CHORI-230 female Brown Norway rat BAC Library (BACPAC Resources Center, Children’s Hospital Oakland Research Institute) by searching the rat BAC ends database at the National Center for Biotechnology Information. The DNA fragments encoding firefly luciferase (fLuc) and human oestrogen receptor ligand binding domain (hERL) were introduced at the initiation methionine codon in exon 1 of the rat NG2 BAC clone using a Red/ET Quick & Easy BAC modification Kit (Gene Bridges). The recombinant BAC construct was linearized by PI-SceI digestion and purified by pulse field gel electrophoresis. The purified constructs were microinjected into Wistar rat fertilized eggs. The fertilized eggs were implanted into pseudopregnant Wistar rat females. Transgenic (Tg) founders were identified by Southern blot analysis of genomic DNA derived from tail biopsies. The Tg rats expressed fLuc and hERL in NG2 cells for dual-modal bioluminescence and positron emission tomography (PET) imaging of NG2 cells, respectively. In this study, the Tg rats were used for *in vivo* bioluminescence imaging. We confirmed that expression of hERL in NG2 cells has no effect on hair cycle in Tg rats under physiological conditions.

All (normal Wistar rats, Tg rats, and wild type (WT) littermates) rats were housed under controlled temperature (20 ± 2 °C), humidity (50 ± 10%) and maintained under 12-hour light/dark cycles with free access to food and water. All experimental protocols were approved by the Ethical Committee on Animal Care and Use of RIKEN and performed in accordance with the Principles of Laboratory Animal Care (NIH publication No. 85-23, revised 1985).

### Genotyping

PCR genotyping for the transgenic offspring was performed using specific primer sequences for fLuc transgene: (forward 5′-TCAAAGAGGCGAACTGTGTG-3′, reverse 5′-GGTGTTGGAGCAAGATGGAT-3′). Amplified DNA fragments were separated on 1.0% agarose gels via electrophoresis and stained with ethidium bromide.

### Analysis of tissue mRNA expression

Total RNA was extracted from brain and skin tissues using an ISOGEN kit (NIPPON GENE, Tokyo, Japan). RNA (1 μg) samples were reverse-transcribed into cDNA using the PrimerScript RT reagent Kit with gDNA Eraser (Takara Bio, Otsu, Japan). cDNA samples from each tissue were amplified using the KAPA SYBR FAST Universal qPCR Kit (Kapa Biosystems, Wilmington, MA, USA) on a Thermal Cycle Dice Real Time system TP800 (Takara Bio, Otsu, Japan). PCR primers used for the detection of fLuc and NG2 were as follows: forward (5′-ACAACCGCGAAAAAGTTGCG-3′), reverse (5′-TGCGTCGAGTTTTCCGGTAAG-3′) for fLuc and forward (5′-ACAACACCACACCCAAGACAC-3′), reverse (5′-TCAAGCTGCATCCATACTG-3′) for NG2.

### Immunohistochemistry

Rats were deeply anesthetized using isoflurane and perfused transcardially with 4% formaldehyde buffered with 0.01 M phosphate-buffered saline (PBS; pH 7.4). Skin samples were removed, postfixed in 4% formaldehyde solution in 0.01 M PBS at 4 °C overnight, and immersed in 30% sucrose solution. Skin sections (40-μm thickness) were prepared using a cryostat and collected as free-floating sections. For double-immunostaining, the skin sections were incubated with several primary antibodies at 4 °C for 12–18 h; these antibodies were as follows: monoclonal mouse anti-NG2 IgG (1: 200, Millipore), polyclonal rabbit anti-NG2 IgG (1: 200, Millipore), polyclonal rabbit anti-Ki67 IgG (1: 1,000, NovoCastra), polyclonal rabbit anti-Lgr5 (GPR49) IgG (1: 100, Abcam), polyclonal rabbit anti-CD34 IgG (1: 150, Abcam), polyclonal rabbit anti-cytokeratin 15 (CK15) IgG (1: 100, Abcam), polyclonal rabbit anti-Gli1 IgG (1: 200, Abcam), polyclonal goat anti-luciferase IgG (1: 50, Promega). After washing for 30 min with PBS containing 0.3% Triton-X100 (PBST), skin sections were incubated with the appropriate secondary antibodies conjugated with either Cy2 or Cy3 (1: 200, Jackson ImmunoResearch) at 4 °C for 3–4 h, and washed with PBST for 30 min. The stained sections were mounted with Hoechst dye 33258 (Nacalai Tesque Inc.) and examined using a confocal laser microscope (Digital Eclipse C1; Nikon).

### Bioluminescence imaging

For *in vivo* bioluminescence imaging, NG2-fLuc Tg rats were anesthetized with pentobarbital (50 mg/kg) and were carefully removed their hair in the back skin by using only electric clippers or both clipper and depilatory creams without some skin damage. After shaving, the rats placed in a light-tight imaging chamber equipped with a CCD camera of bioluminescent imaging system (IVIS kinetics, Caliper Life Sciences, Hopkinton, MA, USA) and then, were intravenously injected with D-luciferin at a dose of 30 mg/kg (Promega) through intravenous cannulas. In bioluminescence imaging, intraperitoneal injection of D-luciferin is most commonly used. However, in the present study, D-luciferin injection was administered via intravenous injection, as this enables the generation of higher bioluminescent signals relative to D-luciferin injection via the intraperitoneal route. Accordingly, bioluminescence signals from the dorsal skin surface of intravenously injected Tg rats receiving D-luciferin, at 30 mg/kg, were higher than those from intraperitoneally injected rats receiving the same dose. Bioluminescence images were captured for 20 minutes after injection of D-luciferin by the minute. The bioluminescence signal intensities from each region of the rats were quantified by region of interest (ROI) analysis using Living Image analysis software (Xenogen, Alameda, CA). ROIs were defined on the dorsal skin, from the upper limb to the upper end of the femur in the rostral-caudal direction. Time-intensity curves showed that bioluminescence signals peaked at 5 minutes after intravenous injection of D-luciferin. The signal intensity in the ROI area was calculated as the average value for 3 minutes before and after the maximum value and expressed in photons/sec/cm^2^/sr. Background images were collected daily and background subtractions were automatically calculated by the software.

For *ex vivo* bioluminescence imaging, the rats were sacrificed at 5 min after luciferin injection. Then, each tissue was immediately removed and placed on a heated stage in a chamber. The bioluminescence images were taken at 5–10 min after injection of D-luciferin, using the IVIS imaging system.

### Statistical analysis

Data from each animal were presented as means ± SD. Data for all experiments were analysed using Mann-Whitney test (2-tailed nonparametric analysis). A statistical significance threshold was assumed at P < 0.05.

## Electronic supplementary material

Supplemental Data
