# Supplementary material for: In vivo monitoring of hair cycle stages via bioluminescence imaging of hair follicle NG2 cells
Source: Sci Rep. 2018 Jan 10;8:393. doi: 10.1038/s41598-017-18763-3 (PMC5762894; doi:10.1038/s41598-017-18763-3)
Supplement: Supplementary file 1 — Supplemental Data [file 41598_2017_18763_MOESM1_ESM.pdf]

Supplementary Information

In vivo monitoring of hair cycle stages via bioluminescence imaging of hair follicle NG2 cells

Yasuhisa Tamura*, Kumi Takata, Asami Eguchi, and Yosky Kataoka

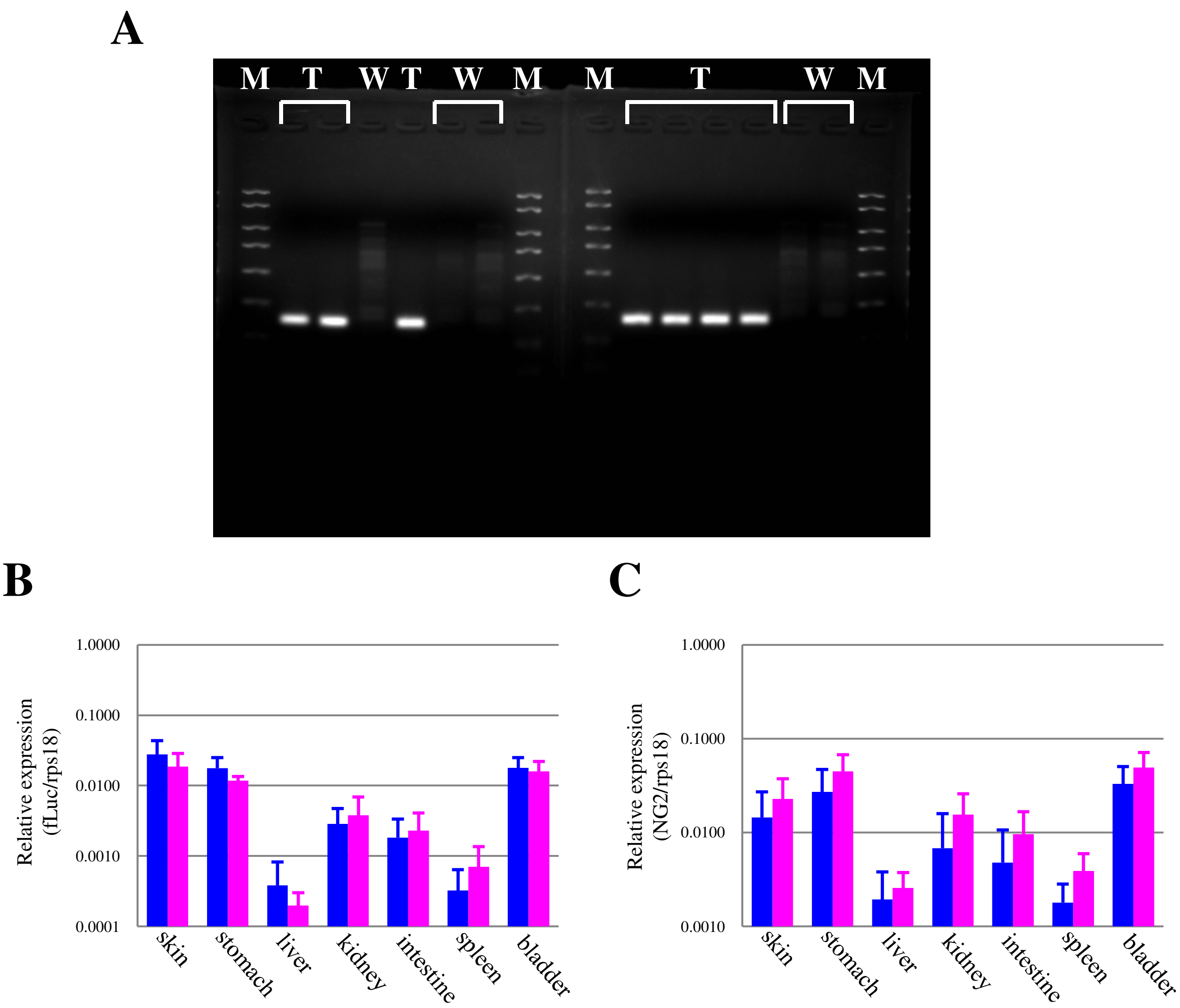

Supplemental Figure S1

Characterization of rNG2-fLuc transgenic (Tg) rats. (A) Identification of Tg rats by PCR analysis; M, DNA marker; W, wild type rats; T, transgenic rats. (B-C) Expression of the fLuc reporter gene (B) and NG2 (C) in several tissues of 8-week-old NG2-fLuc Tg rats during the telogen phase. Real-time PCR analysis was performed for quantification of the expression of both genes in the brain, skin, stomach, liver, kidney, intestinal tract, and bladder of male (blue bars; N = 5) and female (pink bars; N = 5) Tg rats. The expression of the genes was normalized to that of rps18.

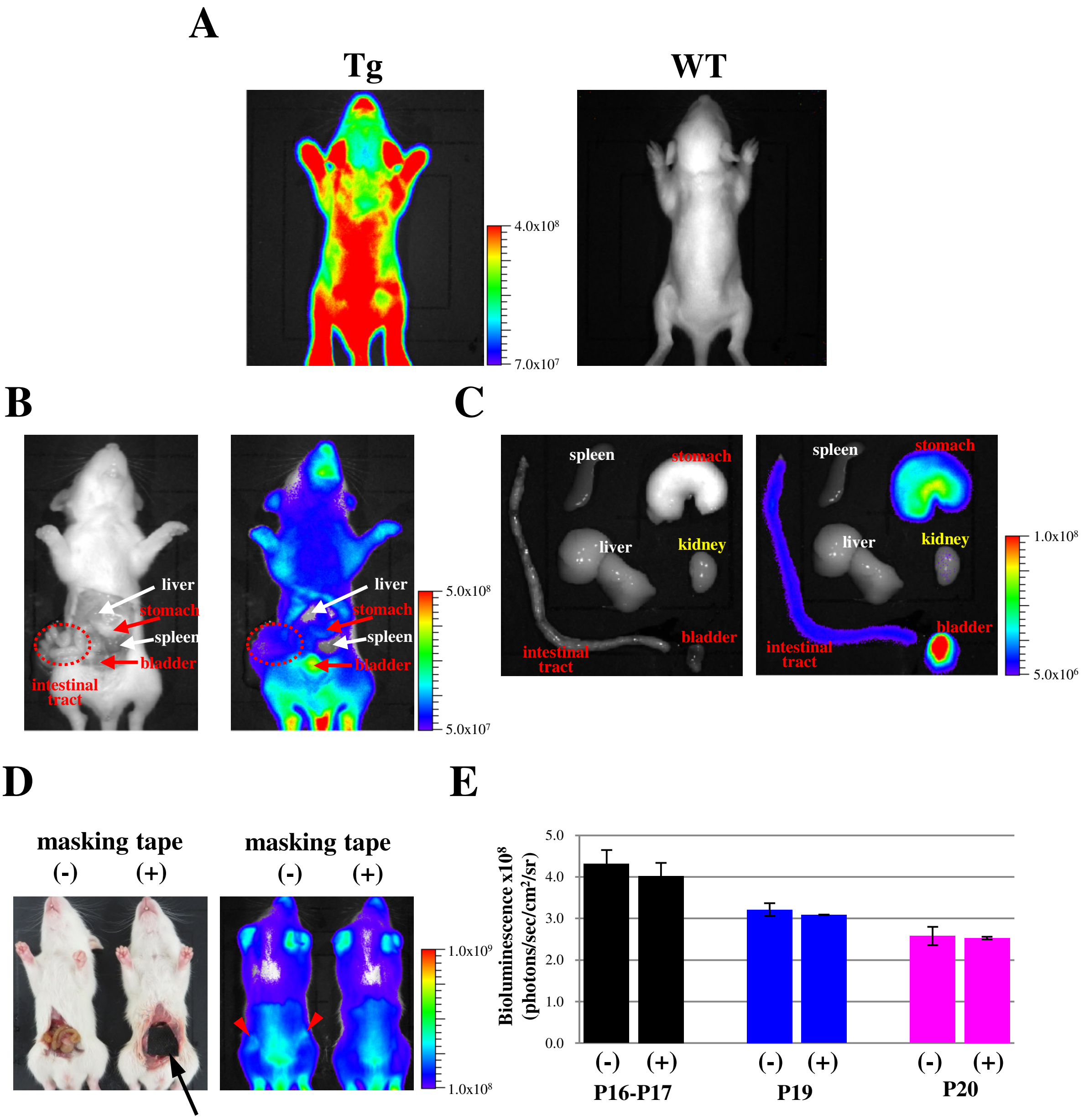

Supplemental Figure S2

In vivo bioluminescence imaging of NG2-fLuc Tg infant rats. (A) Bioluminescence images of Tg (left panel) and WT (right panel) rats at postnatal day 16 (P16). (B) In vivo bioluminescence imaging of abdominal tissues in male P19 Tg rats indicated that bioluminescence was detected in the stomach and intestinal tract (red arrows), but not in the liver and spleen (white arrows). (C) Ex vivo imaging of internal organs of male P19 Tg rats indicated that bioluminescence was detected in the stomach, intestinal tract, bladder, and kidney, but not in the liver and spleen. Bioluminescence signals in the kidney were much weaker than those in the other organs. (D) Comparison of bioluminescence signals from the dorsal surface skin of male P16 Tg rats with (right panel) or without (left panel) masking tape (black arrow), which completely blocked bioluminescence signals from internal organs, including the stomach, liver, spleen, kidney, intestinal tract, and bladder. Bioluminescence signals from kidney (red arrowheads) were weakly detected from the dorsal skin of Tg rats without masking tape whereas these signals were not found in the Tg rats with them. (E) Comparison of bioluminescence signals from the dorsal surface skin between each group in P16-P17 (N = 3 per group, black bars), male P19 (N = 2 per group, blue bars) and female P20 (N = 2 per group, pink bars) Tg rats.

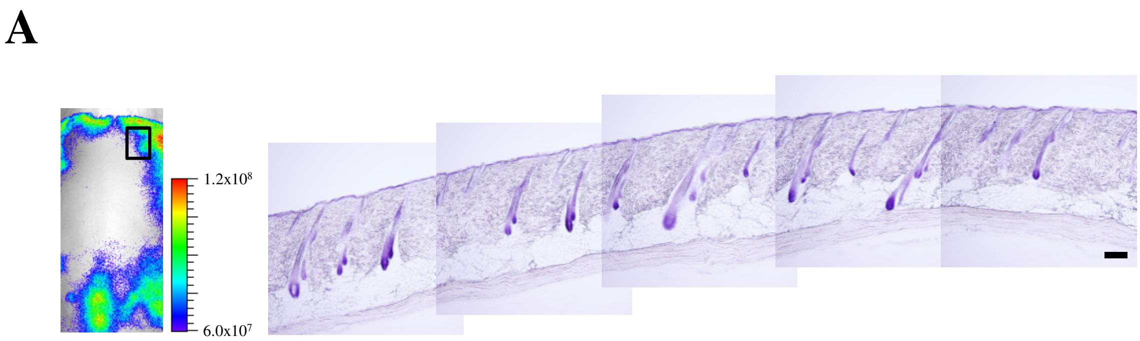

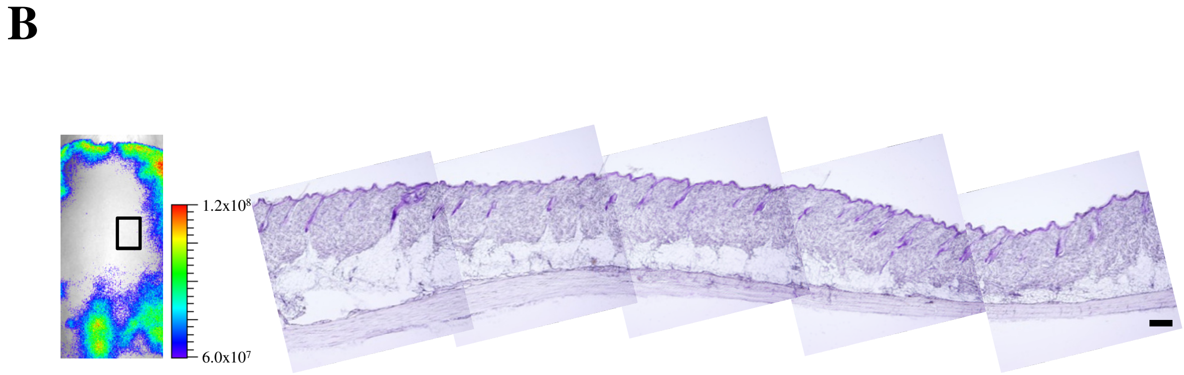

Supplemental Figure S3
Relationship between bioluminescence imaging data and histological staining data for the skin of aged Tg rats; images of haematoxylin staining of the dorsal skin showing the fragmentation of hair cycling domains (A) and the retention of the telogen phase (B) in aged Tg rats. (A) A batch of the hair follicles in the defined rectangle entered into the anagen phase, whereas another batch of the hair follicles in the same rectangle was in the telogen phase in the histologically-staining section. (B) Almost all hair follicles in the defined rectangle showed to be in the telogen phase in the skin section. These stained sections were prepared from the skin of dorsal region indicated with the black rectangle; scale bars, 500 µm.
